# Preterm Birth and Low Birth Weight after *In Utero* Exposure to Antiretrovirals Initiated during Pregnancy in Yaoundé, Cameroon

**DOI:** 10.1371/journal.pone.0150565

**Published:** 2016-03-21

**Authors:** Anne Esther Njom Nlend, Annie Nga Motazé, Suzie Moyo Tetang, Cécile Zeudja, Marcus Ngantcha, Mathurin Tejiokem

**Affiliations:** 1 Department of Pediatrics, National Social Insurance Fund Hospital, Essos Hospital Center, Yaoundé, Cameroon; 2 Independent Consultant, Paris, France; 3 Epidemiology and Public Health unit, Centre Pasteur of Cameroon, Yaoundé, Cameroon; University of Cape Town, SOUTH AFRICA

## Abstract

**Background:**

Effects of antiretroviral therapy (ART) on birth outcomes remain controversial.

**Objective:**

To assess the impact of antenatal exposure to ART on the occurrence of preterm birth (PTB) and low birth weight (LBW).

**Methods:**

A cross-sectional study conducted at the Essos Hospital Center in Yaounde from 2008 to 2011 among HIV vertically exposed infants with two distinct maternal antiretroviral experiences: monotherapy group (Zidovudine, ZDV) and the combination ART group (cART). Mothers already receiving cART before pregnancy were ineligible. In both groups, events of PTB (<37 weeks) and LBW (<2,500g) were analyzed using univariate and multivariate logistic regression; with p<0.05 considered statistically significant.

**Results:**

Of the 760 infants, 481 were born from cART-exposed mothers against 279 from maternal-ZDV. Median maternal CD4 count was 378 [interquartile range (IQR): 253–535] cells/mm^3^. Median duration of ART at onset of delivery was 13 [IQR: 10–17] weeks. In the cART-group, 64.9% (312/481) of mothers were exposed to Zidovudine/Lamuvidine/Nevirapine and only 2% (9/481) were on protease inhibitor-based regimens. Events of PTB were not significantly higher in the cART-group compared to the ZDV-group (10.2% vs. 6.4% respectively, p = 0.08), while onsets of LBW were significantly found in the cART-group compared to ZDV-group (11.6% vs. 7.2% respectively, p = 0.05). Other factors (parity, maternal age at delivery or CD4 cell count) were not associated with PTB.

**Conclusion:**

cART, initiated during pregnancy, would be an independent factor of LBW. In the era of option B+ (lifelong ART to all HIV-pregnant women), further studies would guide towards measures limiting onsets of LBW.

## Introduction

During the last decade, efforts in preventing mother-to-child transmission (PMTCT) of HIV have significantly reduced new HIV pediatric infections worldwide. Of note, antiretroviral drugs, and especially combination antiretroviral therapy (cART), have minimized the risks of HIV vertical transmission (during pregnancy, delivery and breastfeeding) to almost zero in developed countries [1–3]. Furthermore, current promising PMTCT progress in developing countries makes elimination of mother-to-child transmission of HIV (eMTCT) a reality in resource-limited settings [4, 5].

In spite of the great achievements with PMTCT interventions, the safety of cART on birth outcomes remains questionable. Indeed, there are conflicting evidences on the increasing rate of preterm birth (PTB) and/or of low birth weight (LBW) following prenatal antiretroviral exposure [6, 7]. Though available data were mostly from the developed world, some recent reports from sub-Saharan Africa (SSA) showed negative effects following antenatal exposure to ART, among which PTB and LBW were frequently addressed [8–10]. Interestingly, monotherapy (i.e. option A) and cART (option B or B+) used during pregnancy in SSA mostly consist of reverse transcriptase inhibitors, which are drugs with lesser antenatal impairments, compared to protease inhibitor-based regimens commonly used in developed countries [11]. Thus, understanding the impact of commonly used antiretrovirals on birth outcomes in SSA would serve in designing interventions aiming at: (a) continuing current ART during pregnancy without further interventions, (b) continuing current ART during with specific monitoring measures, or (c) switching from current ART to potential regimens with safer birth outcomes. Such considerations are of utmost importance in SSA countries whereby HIV-infection during pregnancy is consistent, in the frame of PMTCT uptake [3].

Cameroon is a SSA country experiencing a generalized HIV epidemiology with almost a doubled rate among women (5.9%) [5]. Specifically, 7.8% of the subpopulation of pregnant Cameroonian women would be living HIV [4], suggesting considerable number of antenatal ART exposure in the context of PMTCT interventions, and the country’s position among the top 22 with the highest global burden in HIV vertical transmission [3].

**Fig 1:**
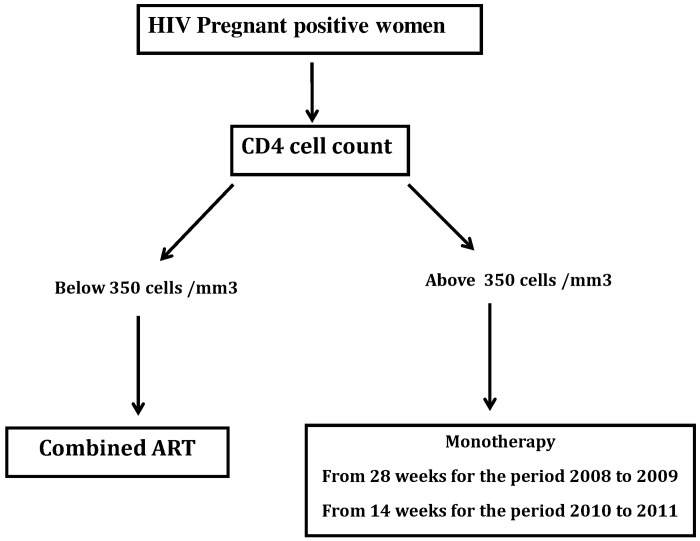
Algorithm for ARV eligibility and type of PMTCT regimens at Essos Hospital Centre from 2008 to 2011, Cameroon.

From 2008–2011, national PMTCT guidelines recommended option A which includes PMTCT prophylaxis with Zidovudine-monotherapy based on the CD4 cell count >350/mm^3^ was prescribed from 28 weeks of pregnancy up to delivery from 2008 to 2009, and later on from 14 weeks up to delivery from 2009–2011; meanwhile pregnant women with CD4 cell count ≤350/mm^3^ were eligible for lifelong cART consisting of Zidovudine (ZDV), Lamivudine (3TC) and Nevirapine (NVP) at any moment during pregnancy (Fig 1) [7]. Current progress is towards a wider implementation of PMTCT option B+, following the global plan in eMTCT with universal access to option B+ by 2017 [3]. Such achievements would lead to virtual elimination of new HIV pediatric infections, implying more antenatal ART exposure with potentials of poor birth outcomes in terms of PTB and LBW [3]. Thus, as Cameroon is effectively transitioning to option B+, we sought to assess the impact of antiretroviral exposure during pregnancy on poor birth outcomes (PTB and LBW), by comparing ZDV-monotherapy versus cART.

## Methods

### Study design and populations

Using a retrospectively designed investigation, we conducted a comparative and cross-sectional assessment of birth outcomes according antiretroviral exposure (ZDV-monotherapy versus cART) among PMTCT-enrolled mother-child pairs at the Essos Hospital Centre (EHC), a referral hospital belonging to the National Social Insurance Fund in Yaoundé, Cameroon. The study-reporting period ranged from 2008 through 2011.

### Description of study site

The EHC is an Approved Treatment Centre for ART in Cameroon, offering all services for infants and adults within the health district area of Djoungolo, in Yaounde. The EHC also serves as a reference center for PMTCT program in the health district, with regular updates in the implementation of PMTCT guidelines according national policies. Briefly, PMTCT program was launched at the EHC in 2008 and involved 25 health facilities, whereby HIV-infected pregnant women received are referred to the EHC for free CD4 cell count and MTCT prophylaxis. All HIV-infected antenatal care (ANC) attendees are monitored by Obstetricians assisted by Midwives based on mother age, gestational age, parity, absolute lymphocytes CD4 cell count, antiretroviral regimen and duration on ART. Gestational age was assessed based on the last menstrual period associated with fundal height and ultrasonography whenever necessary. HIV-infected ANC attendees are advised to deliver in any facility of the Djoungolo health district network; to ease referral of HIV vertically exposed newborns at the EHC within ten days following birth. At registration, neonatal parameters are collected from the maternal medical records: date of birth, gender, term at delivery, and birth weight.

Practically, about 30 to 40 new women are registered for antenatal care on a daily basis, ARV drugs were dispensed on a monthly basis during pregnancy to ensure adherence and therapeutic monitoring, and about 20 HIV-exposed newborns are enrolled in the PMTCT-cascade care on a monthly basis.

### Data abstraction

**Fig 2:**
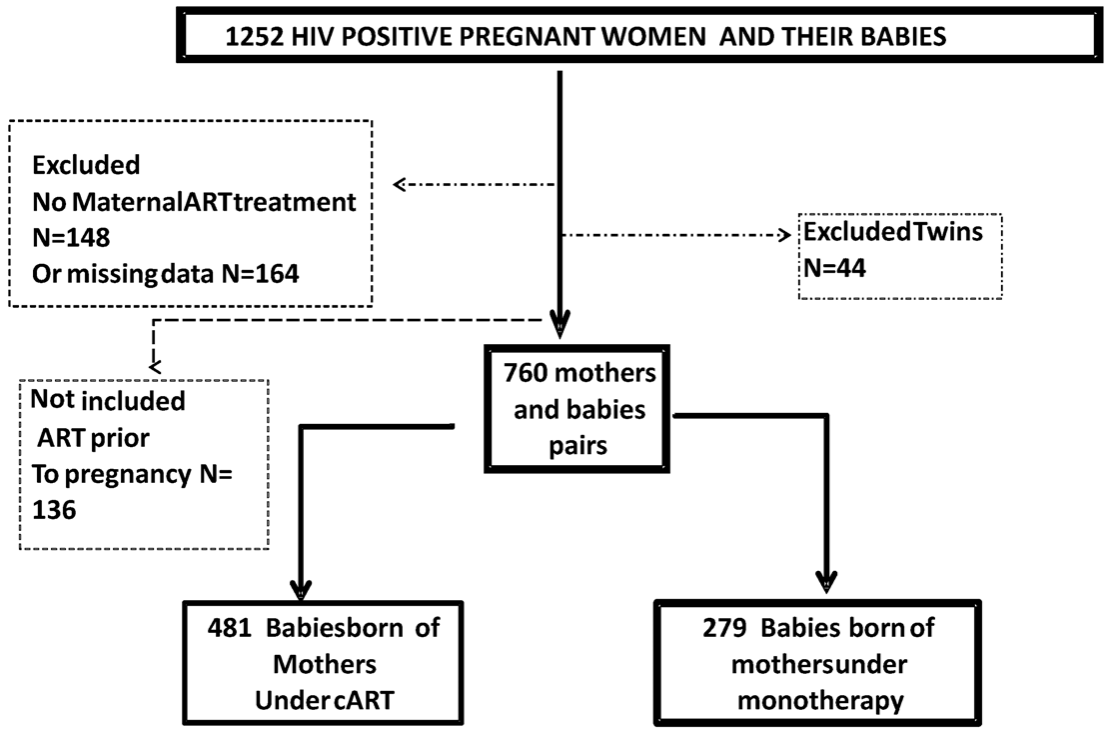
Flowchart of the HIV-positive mothers enrolled in the study, 2008 to 2011, Cameroon.

Data from HIV-infected mothers and their babies routinely monitored in the PMTCT program were abstracted from the register for the period of 2008 through 2011 (Fig 2). HIV-infected pregnant women on cART before pregnancy, or without ANC exposure to antiretroviral drugs, or without information on birth weight or gestational age, were not eligible for the study. Overall, 1252 mother/baby pairs were identified from the EHC register; among them 148 HIV-infected mothers had missed ART and 164 had incomplete data, and 136 were under ART prior to pregnancy, and 44 babies were twins. In the final dataset, 760 files of HIV-infected mothers and their babies were included for analysis and interpretation.

### Data interpretation

Primary outcomes were PTB and LBW. Preterm was defined as babies born alive before 37 weeks of pregnancy. LBW was defined as a birth weight <2,500g regardless of gestational age.

### Statistical analysis

Dependent variables were low birth weight and prematurity. Independent variables were: gestational term at ART initiation, moment of ART initiation, type of antiretroviral regimen, number of CD4 T cells, maternal age and parity. Chi-square and Student tests were used to analyze qualitative and quantitative variables respectively. Univariate and multivariate regression were used for covariates with PTB and LBW. Due to the limited number of independent variables (n = 4), there were all considered in multivariate analysis. Statistical analyses were performed using Stata / IC12.0 Software (College Station, Tx: stataCorp LP), with p-value ≤0.05 considered statistically significant.

### Ethical considerations

Ethical approval was obtained from the Institutional Review Board of the National Social Insurance Fund Hospital. At enrollment for PMTCT intervention, a verbal informed consent was obtained from each mother on the possible use data and reported in the medical file. Data were processed using unique identifiers for purpose of confidentiality, and the data were encrypted for protected.

## Results

### Characteristics of mother-baby pairs

In the total of 760 mother-baby pairs, 481 mothers initiated cART during ANC and 279 received ZDV-monotherapy (Fig 1). These two groups of mother-baby pairs had similar profile in terms of maternal age (26 years versus 27 years, p = 0.07) and parity (1.0 versus 1.0, p = 0.20). Not surprisingly, median CD4 cell count was lower in the group of mothers exposed to cART (302/mm3, Interquartile range [IQR]: 202–416) compared to those under ZDV- monotherapy (533/mm^3^, [IQR: 435–679], p = <0.001), as shown in Table 1.

**Table 1:** Description and comparison of maternal and infants characteristics according to type of antiretroviral exposure during pregnancy, 2008 to 2011, Cameroon.

HIV infected pregnant mothers							
	Exposed to ZDV-monotherapy (N = 279)			Exposed to combination ART (N = 481)			p-value
	Mean	Median	IQR	Mean	Median	IQR	
Maternal age (years) (n = 5)	26.5	26.0	22.0–30.0	27.3	27.0	23.0–31.0	0.07
Parity (n = 87)	1.5	1.0	1.0–2.0	1.4	1.0	1.0–2.0	0.20
CD4 T lymphocyte count (cell/mm^3^)	577.8	533.0	435.0–679.0	322.3	302.0	202.0–416.0	<0.001
Duration on antiretroviral drugs (weeks)	11.5	12.0	8.0–15.0	14.3	14.0	10.0–19.0	<0.01
Birth weight (g)	3209	3200	2900–3500	3113	3150	2800–3500	0.02
Gestational age at delivery (weeks)	39.1	40.0	38–40	39.0	39.0	38–40	0.41

Legend. IQR: interquartile range; n: missing; ART: antiretroviral therapy; ZDV: Zidovudine.

Among mothers exposed to cART during pregnancy, 98% (472/481) were on non-nucleoside reverse-transciptase inhibitor (NNRTI)-based regimens and 2% (9/481) on protease inhibitor (PI)-based regimens. Median duration of antiretroviral exposition was lower in the ZDV-monotherapy group (12 weeks [IQR: 8–15]) as compared to the cART group (14 [IQR: 10–19]); p<0.01.

### Prevalence of PTB and LBW, and associated factors

Overall, the prevalence of PTB was 8.8%, 95% confidence interval (CI): 6.9–11.1, without a significant difference between cART and ZDV-monotherapy groups (10.2% vs 6.4%; p = 0,08).

Of note, HIV-infected mothers exposed to cART had not more premature babies than those exposed to ZDV-monotherapy neither in univariate (OR: 1.6 [95% CI: 0.9–2.9];p = 0.08) nor in multivariate analysis (OR: 1.9 [95% CI: 0.9–3.7];p = 0.06) (Table 2).

**Table 2:** Bivariate and multivariate analysis between maternal characteristics and premature birth, 2008 to 2011, Cameroon.

Maternal characteristics		Premature birth						
		Bivariable					Multivariable	
		N = 760	n = 67	% 8.8	OR [95%CI]	p-value	aOR [95% CI]	p-value
**CD4 T Lymphocytes (cells/mm**^**3**^**) (n = 760)**	≥350	435	35	8.0	1 (ref)	0.39	1 (ref)	0.66
	<350	325	32	9.8	1.2 [0.7–2.1]		0.9 [0.4–1.5]	
**Antiretroviral regimen (n = 760)**	Combination ART	481	49	10.2	1.6 [0.9–2.9]	0.08	1.9 [0.9–3.7]	0.06
	ZDV-Monotherapy	279	18	6.4	1 (ref)		1 (ref)	
**Maternal age (years)**	<21	61	6	9.8	1.1 [0.4–2.7]	0.84	0.9 [0.3–2.4]	0.88
	21–35	649	58	8.9	1 (ref)		1 (ref)	
	>35	45	3	6.7	0.7 [0.2–2.4]		0.8 [0.2–2.6]	
**Parity (n = 673)**	Multiparous	511	46	9.0	1 (ref)	0.31	1 (ref)	0.34
	Primiparous	162	19	11.7	1.3 [0.8–2.4]		1.3 [0.7–2.4]	

aOR: adjusted Odd Ratio

cOR: crude Odd Ratio

Ref: reference

CI: Confidence Interval

ART: Antiretroviral therapy

ZDV: Zidovudine

The prevalence of LBW was 10.0% (95% CI: 7.9–12.4), with a significantly higher rate in babies exposed to cART compared to those exposed to monotherapy (11.6% vs 7.2%, p = 0.05). Similarly, LBW was associated significantly associated with mothers receiving cART (1.7 [1.0–2.9], p = 0.05) compared to those on monotherapy.

According to maternal age during pregnancy, mothers aged less than 21 years had a higher likelihood for LBW compared to mothers aged 21 to 35 years (OR: 2.2 [1.1–4.4]). Following adjustment in multivariable analysis, cART remained as an independent factor associated to LBW (adjusted OR: 1.8 [1.1–3.2] p = 0.03) (Table 3).

**Table 3:** Bivariate and multivariate analysis between maternal characteristics and low birth weight, Essos Hospital Center, 2008 to 2011, Cameroon.

Maternal characteristics		Low birth weight						
		Bivariable					Multivariable (n = 755)	
		N = 760	n = 76	% 10.0	OR [95%CI]	p-value	aOR [95% CI]	p-value
**CD4 T Lymphocytes (cells/mm**^**3**^**) (n = 760)**	≥350	435	42	9.7	1 (ref)	0.71		
	<350	325	34	10.5	1.1 [0.7–1.8]			
**Antiretroviral regimen (n = 760)**	Combination ART	481	56	11.6	1.7 [1.0–2.9]	0.05	**1.8 [1.1–3.2]**	**0.03**
	ZDV-Monotherapy	279	20	7.2	1 (ref)		1 (ref)	
**Maternal age (years) (n = 755)**	<21	61	11	18.0	2.2 [1.1–4.4]	0.10	2.4 [1.2–4. 9]	0.06
	21–35	649	60	9.2	1 (ref)		1(ref)	
	>35	45	5	11.1	1.2 [0.5–3.2]		1.3 [0.5–3.4]	
**Parity (n = 673)**	Multiparous	511	49	9.6	1 (ref)	0.22		
	Primiparous	162	21	13.0	1.4 [0.8–2.4]			

## Discussion

Understanding factors associated with poor birth outcomes in HIV vertically exposed babies is of paramount importance in setting-up approaches to limit such events and their potential impaired effects [8, 9]. In the present observational study, initiating cART during pregnancy for HIV PMTCT could predict onsets of LBW among Cameroonian women. Furthermore, as prematurity does not appear as an independent factor according to antiretroviral exposure, specific monitoring of LBW risks during PMTCT interventions should be recommended. Most importantly, such findings are useful to monitor birth outcomes in the frame of current PMTCT option B+ recommendations [1–3, 12].

By reporting a non-significant burden of PTB with cART, our data are inconsistent with previous findings from other African or Asian settings in newborns, while PTB (15% to 30% in previous studies) was reported to be associated with cART compared to monotherapy used mainly for PMTCT purpose [13, 14]. Onsets of PTB appeared more frequent with PI-based regimens [15], suggesting the need of closer obstetrical monitoring of HIV-infected ANC attendees receiving a second-line ART regimen, since the use of PI is known to favor PTB, especially when initiating cART prior to conception or during pregnancy [16–18].

The rate of LBW from our findings (<20%) differ considerably from data reported in Cote d’Ivoire and in India [8, 11, 12], possibly due to higher immunological status among women in our study. In the frame of findings from in Botswana and other African or Latin America settings, immune-compromised pregnant women receiving PMTCT option B+ should thus far be monitored during routine ANC to limit the potentials of foetal growth retardation in RLS [14, 19, 20], in order to achieve similar target performance in the western world [21, 22].

Interestingly, statistical adjustment for maternal age and parity confirmed the association of cART with LBW, but not with prematurity, stresses the need for collaborative interaction between gynecologists, obstetricians and pediatricians to overcome the onset of LBW in RLS [8–10, 23].

Beside LBW and PTB, there are other factors known to potentially affect fetal growth (maternal hypertension, and mother’s nutritional status) [24, 25]. Furthermore, HIV disease progression has also been identified as a predictive factor of impaired birth outcomes in several settings through placental infection, malaria co-infection, etc [20–22, 26–29]. All these factors, not covered in our present study, could have potentially affected our findings, thus calling for a more comprehensive investigation. Though not feasible in our context, combining the two study outcomes into birth weight Z-score adjusted for gestational age at delivery and gender would have been more relevant.

Taking into account the overall benefit of cART in the context of PMTCT option B+ our findings support the safety of currently used ART during pregnancy, as previously postulated from the dream cohort [30].

In an era of scaling-up in PMTCT option B+ implementation in the entire sub-Saharan Africa, there is need to closely monitor the birth outcomes from pregnant women receiving lifelong, with a wider data safety and monitoring to address potential confounders [15, 31, 32]. Therefore, wherever possible, implementing an interventional trial would provide stronger evidence toward a global translational application.

Conclusively, ANC with exposure to cART may be associated to LBW, especially when the mother is less than 21 years. Additional studies should further explore onsets of PTB and other safety features on cART while scaling-up PMTCT.

## Supporting Information

S1 Data (TXT)

S1 Text (DOC)
