# Supplementary material for: Preterm Birth and Low Birth Weight after In Utero Exposure to Antiretrovirals Initiated during Pregnancy in Yaoundé, Cameroon
Source: PLoS One. 2016 Mar 21;11(3):e0150565. doi: 10.1371/journal.pone.0150565 (PMC4801361; doi:10.1371/journal.pone.0150565)
Supplement: S1 Text — (DOC) [file pone.0150565.s002.doc]

Database name : poids_naiss.dta

obs: 760

vars: 8 21 Sep 2015 22:47

size: 10,640

---------------------------------------------------------------------------------------------------

storage display value

variable name type format label variable label

---------------------------------------------------------------------------------------------------

num int %8.0g Identifiant

ptme str1 %9s MTCT prophylaxis (P=prophylaxis ; T=tritherapy)

age_mere byte %8.0g Mother age

parite byte %8.0g Parity (number of deliveries)

poids_naiss int %8.0g Birth weight

cd4_mere int %8.0g Mother CD4 count during pregnancy

deb_ptme byte %8.0g Age of pregnancy at starting of prophylaxis or tritherapy

terme_naiss float %9.0g Age of pregnancy at delivery

---------------------------------------------------------------------------------------------------
